# Differential Expression Analysis Revealing CLCA1 to Be a Prognostic and Diagnostic Biomarker for Colorectal Cancer

**DOI:** 10.3389/fonc.2020.573295

**Published:** 2020-10-28

**Authors:** Fang-Ze Wei, Shi-Wen Mei, Zhi-Jie Wang, Jia-Nan Chen, Hai-Yu Shen, Fu-Qiang Zhao, Juan Li, Zheng Liu, Qian Liu

**Affiliations:** Department of Colorectal Surgery, National Cancer Center/National Clinical Research Center for Cancer/Cancer Hospital, Chinese Academy of Medical Sciences and Peking Union College, Beijing, China

**Keywords:** CRC, diagnostic, biomarker, prognostic model, database

## Abstract

Colorectal cancer (CRC) is a common malignant tumor of the digestive tract and lacks specific diagnostic markers. In this study, we utilized 10 public datasets from the NCBI Gene Expression Omnibus (NCBI-GEO) database to identify a set of significantly differentially expressed genes (DEGs) between tumor and control samples and WGCNA (Weighted Gene Co-Expression Network Analysis) to construct gene co-expression networks incorporating the DEGs from The Cancer Genome Atlas (TCGA) and then identify genes shared between the GEO datasets and key modules. Then, these genes were screened *via* MCC to identify 20 hub genes. We utilized regression analyses to develop a prognostic model and utilized the random forest method to validate. All hub genes had good diagnostic value for CRC, but only CLCA1 was related to prognosis. Thus, we explored the potential biological value of CLCA1. The results of gene set enrichment analysis (GSEA) and immune infiltration analysis showed that CLCA1 was closely related to tumor metabolism and immune invasion of CRC. These analysis results revealed that CLCA1 may be a candidate diagnostic and prognostic biomarker for CRC.

## Introduction

Colorectal cancer (CRC) is the third most common cancer and the second most deadly cancer worldwide (1). The incidence and mortality of CRC continue to increase because of the lack of diagnostic biomarkers and inadequate understanding of the molecular mechanism (2). Detection and monitoring of CRC occurrence and progression are dependent on a combination of radiologic examinations and serum biomarker measurements (3); however, these methods have some limitations. In some cases, the levels of biomarkers do not change. In other diseases, the levels of biomarkers can change (4, 5). In addition, some patients do not undergo colonoscopy because of the discomfort of this procedure (6). In the past few decades, advanced gene microarray and high-throughput sequencing technologies have been used to explore novel gene expression, treatment targets, and pathogenesis in CRC (7).

Robust rank aggregation (RRA) has been utilized in various recent cancer studies to overcome the limitations of substantial interstudy variability and the different statistical analysis methods used with different technological platforms (8, 9). In our study, we used RRA to analyze 10 microarray datasets from the Gene Expression Omnibus (GEO) database and explored data from The Cancer Genome Atlas (TCGA) through WGCNA to identify differentially expressed genes (DEGs). Gene Ontology (GO) and Kyoto Encyclopedia of Genes and Genomes (KEGG) analyses were used to explore the potential functions of these DEGs. We utilized regression analyses and the random forest method to develop and validate the prognostic model. Among the genes included in the model, we used MCC to calculate the top 20 hub genes. We explored biological functions through GO and KEGG analyses and utilized ROC curves to explore diagnostic value. We also explored the relationship between them. Based on the 20 hub genes, we utilized Kaplan-Meier (K-M) analysis to explore relationships with prognosis, and only CLCA1 had a close relationship with prognosis. We continued to explore the potential biological value of CLCA1. In addition, we utilized the online tool TISIDB and R packages to explore the functions of these genes in immunity and performed gene set enrichment analysis (GSEA) to investigate their potential functions in CRC.

## Materials and Methods

### Gene Expression Datasets

All microarray datasets were downloaded from the TCGA and GEO databases. The RNA sequencing data were downloaded from the TCGA database (https://portal.gdc.cancer.gov/), which contained 41 control tissues and 482 CRC tissues with clinical data. Other datasets that satisfied the following criteria were downloaded from GEO (http://www.ncbi.nlm.nih.gov/geo/): 1) Gene expression data in the microarray datasets included data for both control tissues and CRC tissues, and 2) each microarray contained a minimum of 5 tumor and control tissues. According to the above criteria, 10 GEO datasets were incorporated in this study: GSE9348 (10), GSE44076 (11), GSE4183 (12), GSE20916 (13), GSE37364 (14), GSE44861 (15), GSE81558 (16), GSE22598 (17), GSE113513, and GSE110224 (18).

### Identification of Significant DEGs in CRC Samples

We downloaded the series matrix files from GEO and screened them with the R package “limma” for normalization and DEG identification. Then, the RRA method was utilized to integrate the results of these 10 datasets to identify the most significantly upregulated and downregulated genes (**Supplementary file 1**). Genes with an adjusted P value of <0.05 were considered significantly differentially expressed. For TCGA database analysis, we first separated mRNA and lncRNA data and used the R package “edgeR” (19) to identify DEGs. The following criteria were used to select DEGs: |log(foldchange)|>2 and P value<0.01 (**Supplementary file 2**). After obtaining the 2 sets of DEGs, we used the R package “WGCNA” to identify clinical trait-related modules (20). We used the online tool “VENN” (http://bioinformatics.psb.ugent.be/webtools/Venn/) to generate a Venn diagram to identify genes shared between the key modules from the TCGA and GEO datasets (21). We ultimately obtained 129 DEGs.

### GO and KEGG Functional Enrichment Analyses

We conducted GO enrichment analysis using the online tool Database for Annotation, Visualization, and Integrated Discovery (DAVID; https://david.ncifcrf.gov/) (22) and the R packages “digest” and “GOplot”; an adjusted P value of <0.05 was considered statistically significant. For KEGG pathway analyses, we used the R packages “clusterprofiler” (23), “org.Hs.eg.db”, “enrichplot”, and “ggplot2”, with an adjusted P value of <0.05 considered statistically significant. Both GO enrichment and KEGG analysis results were visualized using the R package “GOplot”.

### Hub Genes from the DEG Network

We utilized the online STRING database (https://string-db.org/cgi/input.pl/) to explore connections among the DEGs and visualized these connections by constructing a PPI network with Cytoscape software (version 3.6.1) (24). We utilized cytoHubba MCC to calculate the top 20 hub genes. We analyzed relationships between the 20 hub genes using the R package “psych”.

### Development and Validation of the Prognostic Model

We utilized R (version 3.6.1) to generate a matrix that included the clinical information and DEG expression. We used Cox regression analysis to build the prognostic model using the R package “survival” (25, 26) and online tool “SangerBox”. Then, we utilized the R package “randomForest” to validate the prognostic model through risk score and calculate the accuracy, rrror rate, sensitivity and precision from a confusion matrix. The prognostic model was based on the TCGA database.

### Diagnostic and Prognostic Value of the Hub Genes

We utilized SPSS to explore the diagnostic value of the genes for CRC and K-M analysis to determine the prognostic value. We validated the differential expression levels between control tissue and tumor tissue with the R packages “limma” and “beeswarm” utilized GSE44076. We utilized the Wilcoxon and Kruskal-Wallis tests to explore the relationship between gene expression and clinical features in the TCGA-COAD and TCGA-READ datasets.

### Analysis of the Association of Hub Gene Expression With Tumor-Infiltrating Immune Cell Infiltration

We utilized TISIDB (http://cis.hku.hk/TISIDB/) to explore the relationship between the expression of genes and infiltration of tumor-infiltrating immune cells, including CD4^+^ T cells, CD8^+^ T cells, B cells, neutrophils, monocytes, eosinophils, mast cells, DCs, NKT cells, NK cells, MDSCs, and CD56 cells (27, 28). TISIDB is an online tool that includes genomic, transcriptomic and clinical data for 30 cancer types from the TCGA database.

### GSEA of Hub Genes

We utilized GSEA, which was downloaded from (https://www.gsea-msigdb.org/gsea/msigdb), to explore the functions of the hub genes. We performed GSEA of the hub genes with the R package “clusterprofiler” (29) in data downloaded from the TCGA-COAD and TCGA-READ datasets and divided 482 samples into two groups: high expression and low expression. We utilized “c2.cp.kegg.v6.2.symbols.gmt” for analysis and to select the top 5 genes. Then, we used the R packages “plyr”, “ggplot2”, “grid”, and “gridExtra” to integrate different significant pathways into a single diagram.

### Validation of Protein Expression and Prognostic Value of CLCA1

We utilized GEO online tools PROGgene online database (http://genomics.jefferson.edu/proggene/), The Human Protein Atlas (https://www.proteinatlas.org/), and Kaplan-Meier Plotter (http://kmplot.com/analysis/) to explore the protein expression and prognostic value of CLCA1. The Human Protein Atlas is the online database which provides the distribution of human proteins in tissues and cells, and immunohistochemical techniques are used to examine the distribution and expression of each protein in 48 normal tissues and 20 tumor tissues. Kaplan-Meier Plotter is the online database which including the data from GEO, EGA and TCGA.

## Results

### Identification of the Significantly Differentially Expressed Genes in the Datasets

**Figure 1:**
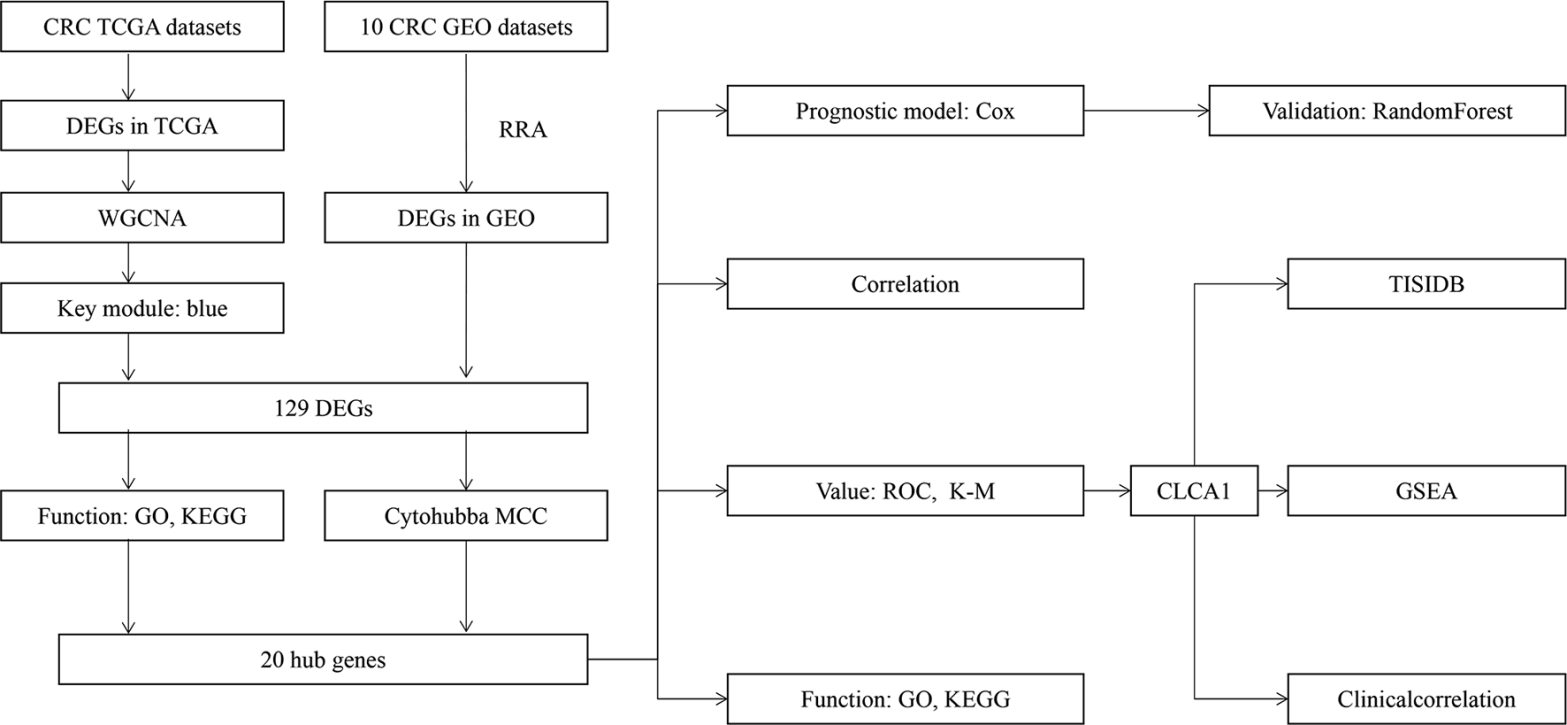
Analysis workflow of this study.

**Figure 2:**
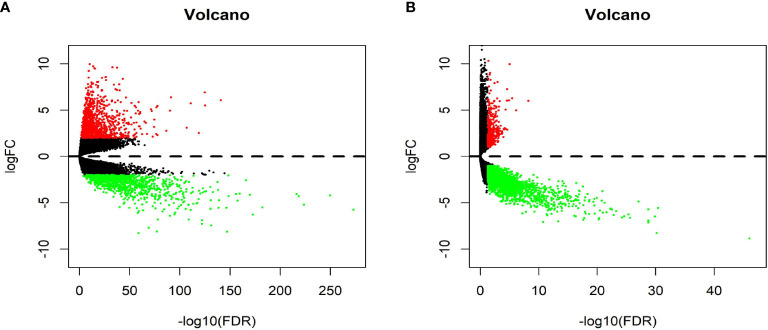
Volcano plot of The Cancer Genome Atlas (TCGA) data. **(A)** Volcano plot depicting the differential expression and distribution of TCGA-COAD data. **(B)** Volcano plot depicting the differential expression and distribution of TCGA-READ data.

**Figure 3:**
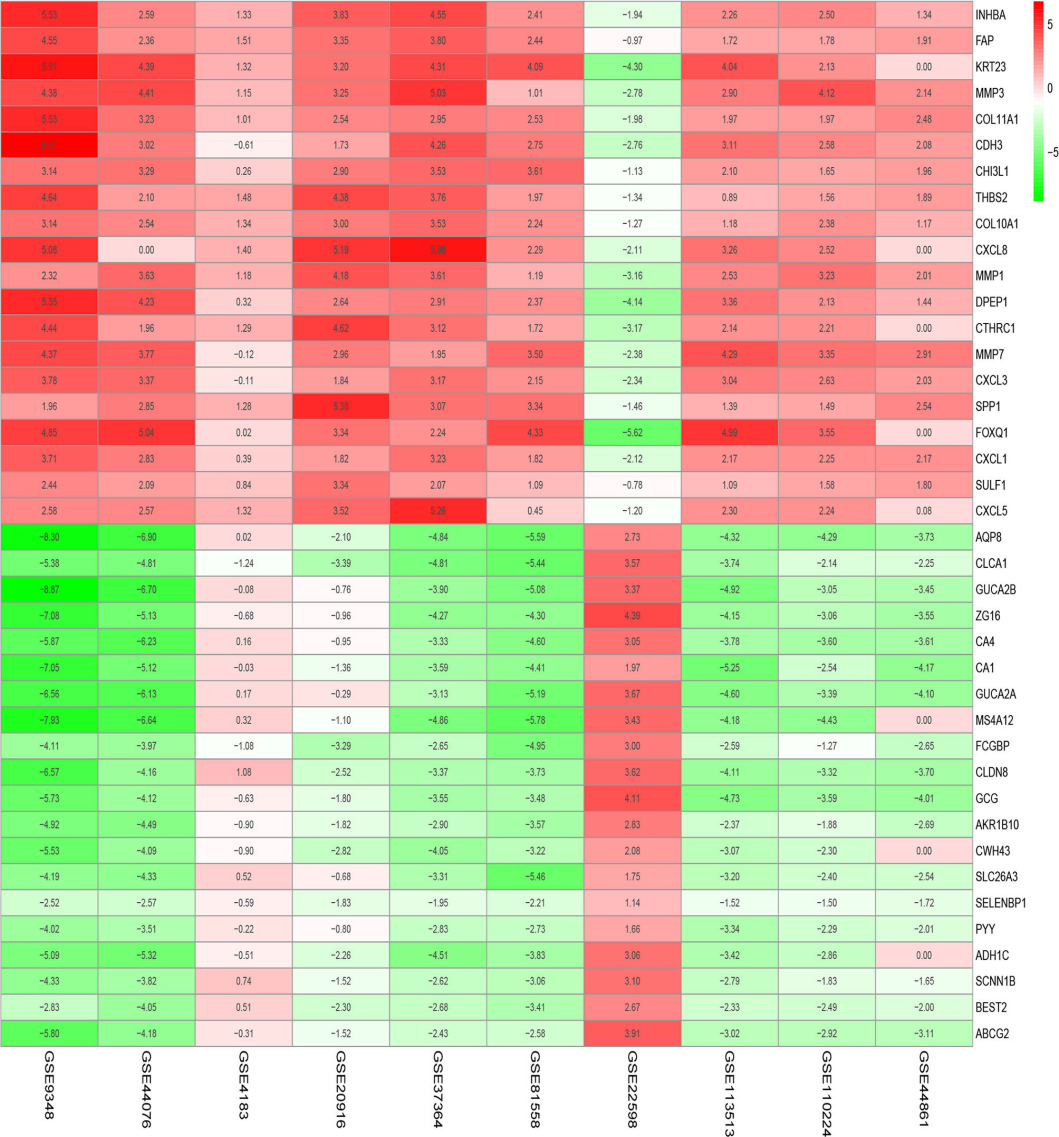
Significantly differentially expressed genes in Gene Expression Omnibus (GEO) datasets by robust rank aggregation (RRA) analysis. These heatmaps show the top 20 downregulated and top 20 upregulated genes. Each column indicates one dataset, and each row indicates one gene. Green indicates downregulation, and red indicates upregulation. The numbers in the heatmap indicate the logarithmic fold changes in the expression of each gene in the dataset.

**Figure 1** shows the workflow of our study. We downloaded CRC samples from the TCGA-COAD and TCGA-READ datasets and identified DEGs between the control tissues and tumor tissues. In total, 2097 genes in the TCGA-COAD dataset and 2887 genes in the TCGA-READ dataset were differentially expressed between tumor and control tissues. The volcano plots of these genes are shown in **Figure 2**. According to the selection criteria for the GEO data, we selected 10 eligible CRC datasets for exploration. The characteristics of all datasets are shown in **Table 1**. RRA analysis of the GEO datasets identified 212 significantly downregulated and 136 significantly upregulated genes. The top 20 downregulated and top 20 upregulated genes are shown in a heatmap (**Figure 3**).

**Table 1:** Characteristics of the datasets.

Dataset	No. of Normal	No. of Tumor	Platform ID	No. of Row Perl Platforms
TCGA-COAD	39	398	RNAseq	17557
TCGA-READ	2	84	RNAseq	17418
GSE9348	12	70	GPL570	54,675
GSE44076	148	98	GPL13667	49,386
GSE4183	15	38	GPL570	54,675
GSE20916	109	36	GPL570	54,675
GSE37364	67	27	GPL570	54,675
GSE44861	55	56	GPL3921	22,277
GSE81558	9	42	GPL15207	49,395
GSE22598	17	17	GPL570	54,675
GSE113513	14	14	GPL15207	49,395
GSE110224	17	17	GPL570	54,675

TCGA-COAD, TCGA-Colon adenocarcinoma; TCGA-READ, TCGA-Rectum adenocarcinoma; GSE, Gene Expression Omnibus Series; GPL, Gene Expression Omnibus Platform.

### WGCNA and Identification of DEGs

**Figure 4:**
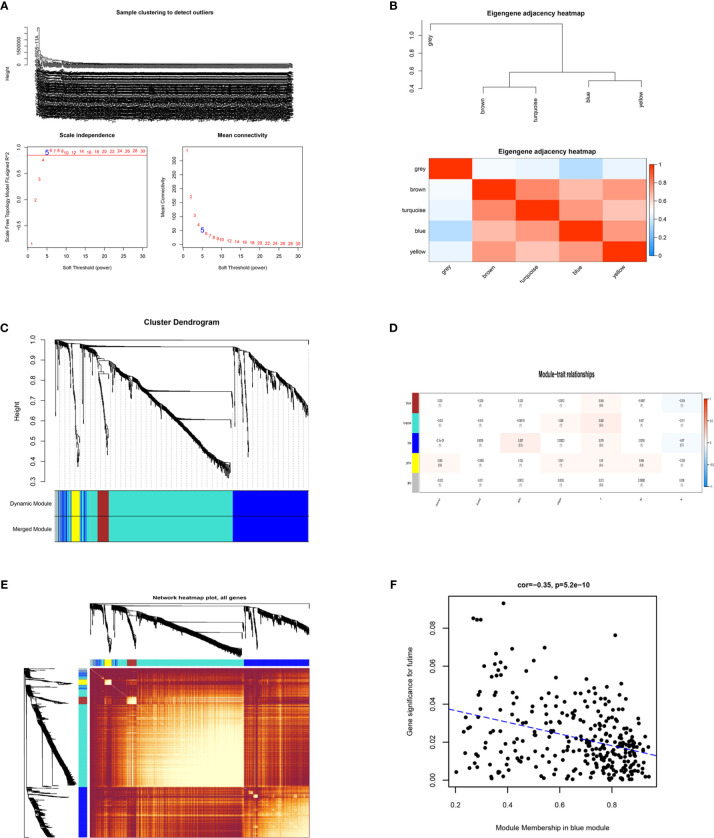
Identification of key modules correlated with clinical traits in the The Cancer Genome Atlas (TCGA)-COAD and TCGA-READ datasets through Weighted Gene Co-Expression Network Analysis (WGCNA). **(A)** Clustering dendrograms of genes. Analysis of the scale-free fit index (left) and the mean connectivity (right) for various soft-thresholding powers. **(B)** Clustering of module eigengenes and a heatmap of adjacent eigengenes. **(C)** Dendrogram of all differentially expressed genes (DEGs) clustered from TCGA based on a dissimilarity measure. **(D)** Heatmap of the correlation between module eigengenes and clinical traits of colorectal cancer (CRC). Each cell contains the correlation coefficient and P value. **(E)** TOM network heatmap of all genes. **(F)** Scatter plot of module eigengenes in the blue module. The genes in the blue module with significance in futime.

**Figure 5:**
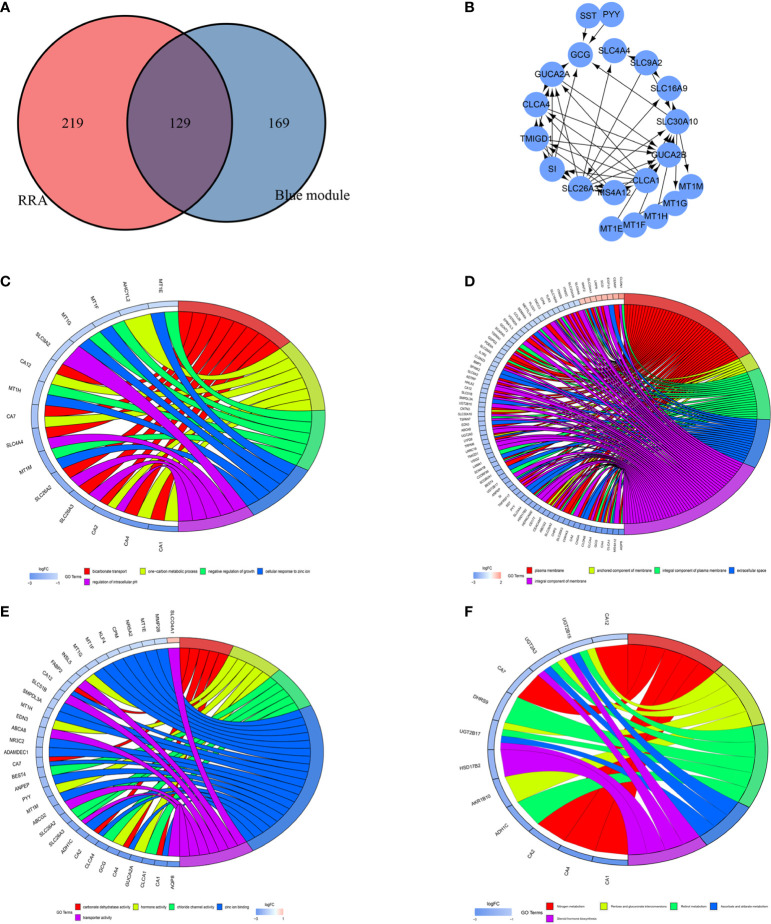
Identification of differentially expressed genes (DEGs) and hub genes. Gene ontology (GO) and Kyoto Encyclopedia of Genes and Genomes (KEGG) analyses of the DEGs. **(A)** Venn diagram showing the numbers of DEGs. Red indicates blue module genes in Weighted Gene Co-Expression Network Analysis (WGCNA) from the TCGA-COAD and TCGA-READ datasets. Blue indicates significantly differentially expressed genes in robust rank aggregation (RRA) analysis of Gene Expression Omnibus (GEO) datasets. **(B)** PPI network of 20 hub genes. **(C)** Chord plot depicting the relationships between the genes and GO biological process (BP) terms. **(D)** Chord plot depicting the relationships between the genes and GO cellular component (CC) terms. **(E)** Chord plot depicting the relationships between the genes and GO molecular function (MF) terms. **(F)** Chord plots depicting the functions of the genes in KEGG pathways.

To identify the key modules most associated with CRC clinical traits, we performed WGCNA on the significant genes in the TCGA-COAD and TCGA-READ datasets (**Figures 4A–E**). Clinical information such as age, TNM grade, and survival time was retrieved from TCGA. By setting a soft-thresholding power of 5 (scale free R^2^ = 0.89), we eventually identified 5 modules. From the heatmap of module-trait correlations, we found that the bule module was the most highly correlated with clinical traits, especially the futime (P=5.2e-10; **Figure 4F**). The blue module contained a total of 299 genes, as shown in **Figure 4F**. We combined the genes from the blue module and the RRA analysis and used Venn diagrams to identify significantly DEGs common to the 2 datasets, as shown in **Figure 5A**. The 129 DEGs were visualized using STRING and Cytoscape software, and CytoHubba MCC was used to calculate the top 20 hub genes, as shown in **Figure 5B**.

### GO and KEGG Enrichment Analyses of DEGs

We used DAVID to explore the main 3 categories of GO enrichment: biological process (BP), cellular component (CC), and molecular function (MF). In the BP category, we explored bicarbonate transport (P=1.62E-08), one-carbon metabolic process (P=1.57E-06), negative regulation of growth (P=6.97E-06), cellular response to zinc ion (P=6.97E-06) and regulation of intracellular pH (P=9.70E-05) **(** **Figure 5C**). In the CC category, we identified plasma membrane (P=7.41E-04), anchored component of membrane (P=0.001050845), integral component of plasma membrane (P=0.007269043), extracellular space (P=0.009771536) and integral component of membrane (P=0.013058288) **(** **Figure 5D**). In the MF category, we explored carbonate dehydratase activity (P=1.81E-06), hormone activity (P=4.20E-04), zinc ion binding (P=7.40E-04), chloride channel activity (P=4.76E-04) and transporter activity (P=0.002384793) (**Figure 5E**). For KEGG pathway analysis, we explored the top 5 pathways that satisfied the criteria of pFilter<0.05 and adjPfilter<1: nitrogen metabolism, pentose and glucuronate interconversions, retinol metabolism, ascorbate and aldarate metabolism, and steroid hormone biosynthesis (**Figure 5F**).

### Biological Value of the Hub Genes

**Figure 6:**
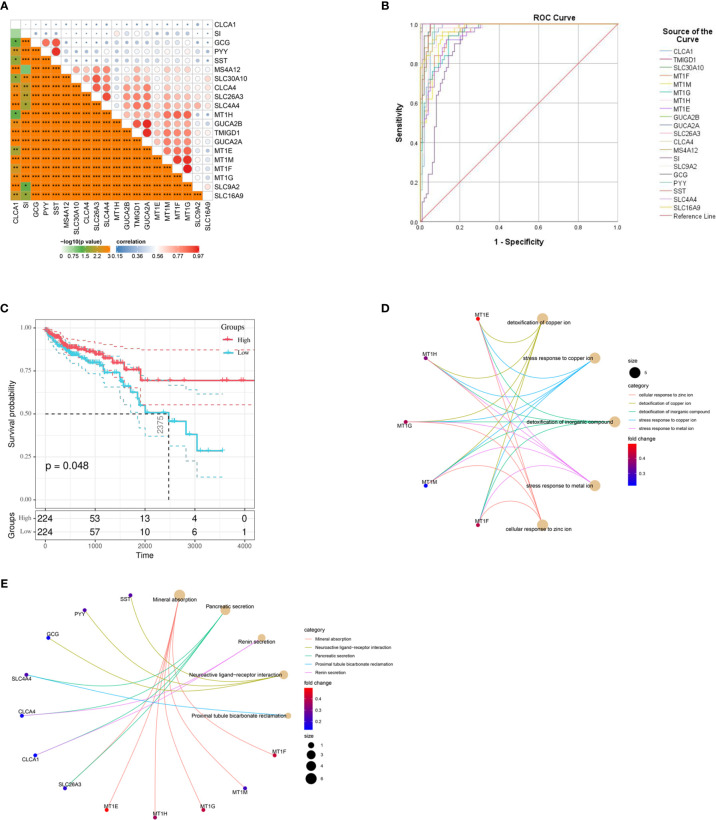
Different values of hub genes and ROC curves of the diagnostic model. **(A)** Hub genes show strong associations with each other. **(B)** ROC curves for the hub genes. **(C)** K-M plot for CLCA1. **(D)** Circo plot depicting the relationships between the hub genes and gene ontology (GO) terms. **(E)** Circo plots depicting the functions of the hub genes in Kyoto Encyclopedia of Genes and Genomes (KEGG) pathways. K-M, Kaplan-Meier.

Through CytoHubba MCC calculation, we obtained 20 hub genes. The 20 hub genes, which are shown in **Figure 6A**, were also closely related to each other. We utilized SPSS to explore their diagnostic value. ROC curve analysis showed that these 20 genes have high diagnostic value for CRC: CLCA1 AUC= 0.959, TMIGD1 AUC= 0.998, SLC30A10 AUC= 0.993, MT1F AUC= 0.933, MT1M AUC= 0.975, MT1G AUC= 0.944, MT1H AUC= 0.947, MT1E AUC= 0.943, GUCA2B AUC= 0.991, GUCA2A AUC= 0.99, SLC26A3 AUC= 0.989, CLCA4 AUC= 0.984, MS4A12 AUC= 0.978, SI AUC= 0.94, SLC9A2 AUC= 0.959, GCG AUC= 0.992, PYY AUC= 0.993, SST AUC= 0.992, SLC4A4 AUC= 0.997, and SLC16A9 AUC= 0.903 (**Figure 6B**). We also explored the prognostic value of the hub genes, and only CLCA1 was closely related to survival time (**Figure 6C**); the other genes are shown in **Supplementary Figure 1**. To further explore the functions of the hub genes, we conducted GO and KEGG analyses. The most significant GO terms for BPs, CCs, and MFs, as well as KEGG pathways, are shown in **Figures 6D, E**.

### Development and Validation of a Prognostic Model Based on the Hub Genes

**Figure 7:**
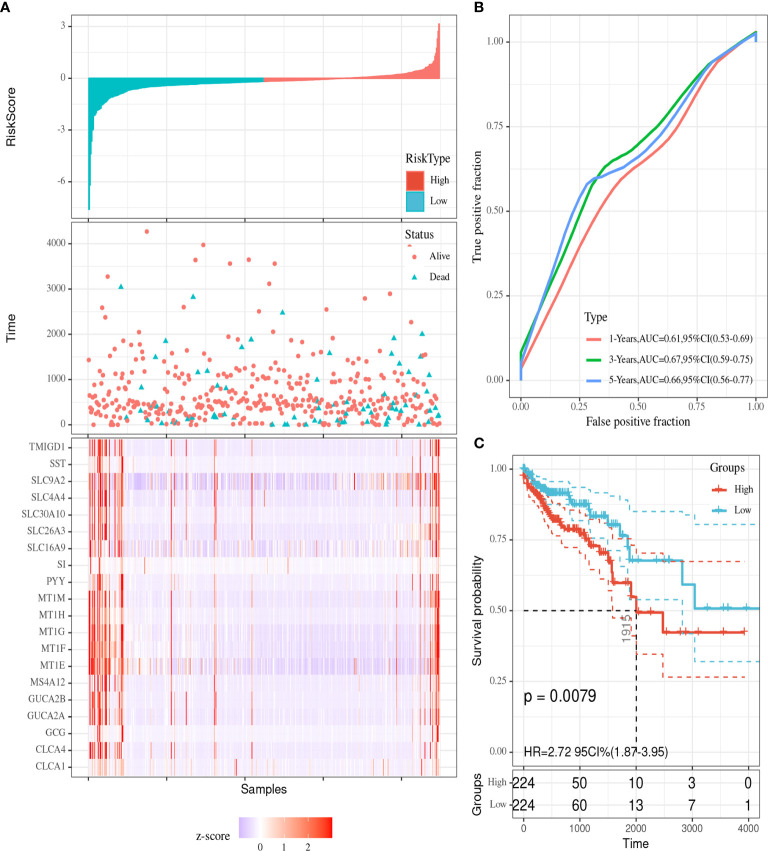
Visualization of the prognostic model. **(A)** The risk score distribution in colorectal cancer (CRC) patients. **(B)** ROC curves for the 1-year, 3-year and 5-year survival rates of CRC patients. **(C)** K-M OS curves for the low-risk and high-risk groups.

We utilized Cox proportional hazards regression analysis of the survival‐related genes to develop the prognostic model (**Figures 7A–C**). According to the prognostic risk score value, CRC patients were divided into a low-risk and a high‐risk group. The risk score distribution was analyzed and is shown in **Figure 7A**. The risk scores reflected the 1-year, 3‐year and 5-year survival rates of CRC patients. The AUCs for 1-year, 3‐year and 5-year survival are shown in **Figure 7B**. K‐M curves were used to show the relationship of the risk score with overall survival (OS) in the low-risk and high-risk groups and verified that a low risk score had a stronger positive association with OS (P=0.0079; **Figure 7C**).

We utilized randomForest to validate the prognostic model. The training group contained 50 died and 263 living patients, and the validation group contained 23 died and 112 living patients. From the confusion matrix, we obtained the following values: accuracy = 79.3%%, error rate = 20.7%, sensitivity = 85%, and precision = 91.1%.

### Assessment of the Clinical Significance of the Hub Genes

**Figure 8:**
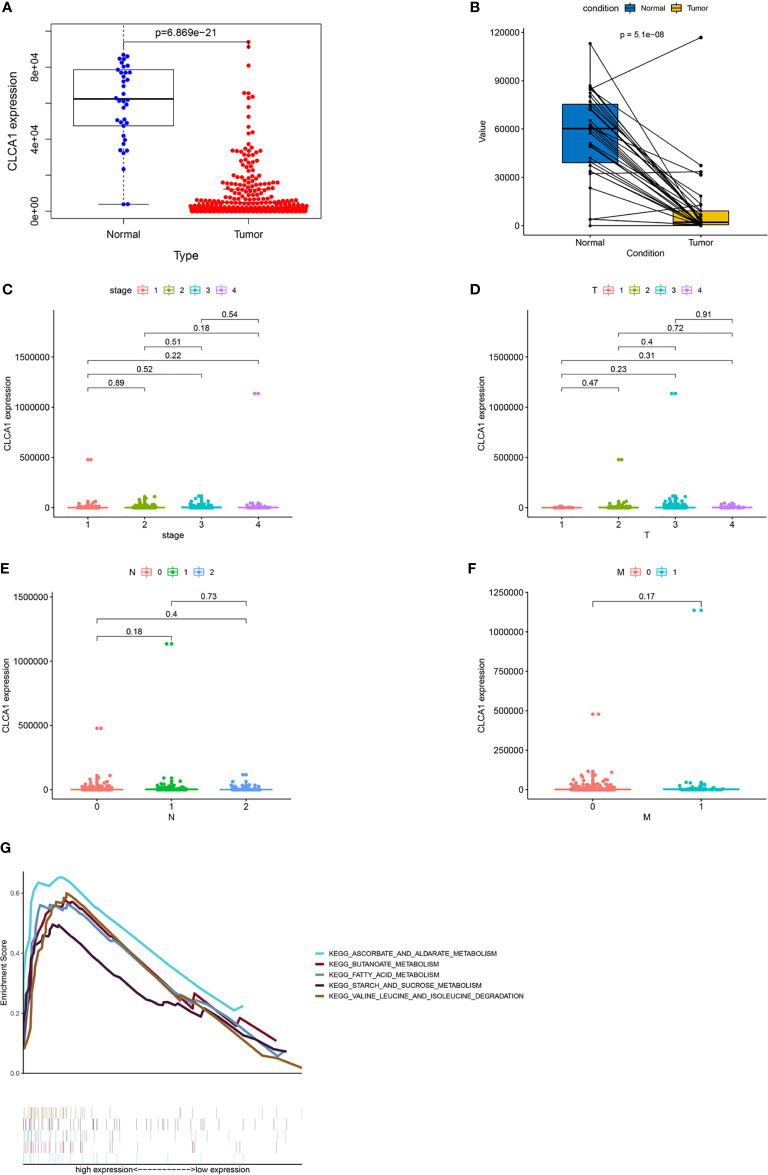
Visualization of correlations between CLCA1 expression levels and clinical features. GSEA of CLCA1. **(A)** Differences in CLCA1 expression between control tissues and CRC tissues. **(B)** Differential expression of CLCA1 between tumor tissues and adjacent tissues. **(C)** Differences in CLCA1 expression between different clinical stages. **(D)** Differences in CLCA1 expression between different T stages. **(E)** Differences in CLCA1 expression between different N stages. **(F)** Differences in CLCA1 expression between different M stages. T, tumor; N, regional lymph node; M, metastasis. **(G)** GSEA for CLCA1. GSEA, Gene set enrichment analysis.

Among the 20 hub genes, CLCA1 was associated with survival time. We explored correlations between gene expression levels (**Figures 8A, B**) and clinical features (**Figures 8C–F**). CLCA1 was downregulated during CRC, and no differences were identified in its expression across different stages and TNM grades. The persistently downregulated expression of CLCA1 underscores its diagnostic effectiveness.

### GSEA for Hub Genes

We performed GSEA to investigate the potential functions of CLCA1 in CRC in the TCGA-COAD and TCGA-READ datasets (**Figure 8G**). The top 5 upregulated pathways in which CLCA1 was enriched included “ascorbate and aldarate metabolism”, “butanoate metabolism”, “fatty acid metabolism”, “starch and sucrose metabolism”, and “valine, leucine, and isoleucine degradation”.

### Relationship of Hub Genes with Immune Infiltration of CLCA1

**Figure 9:**
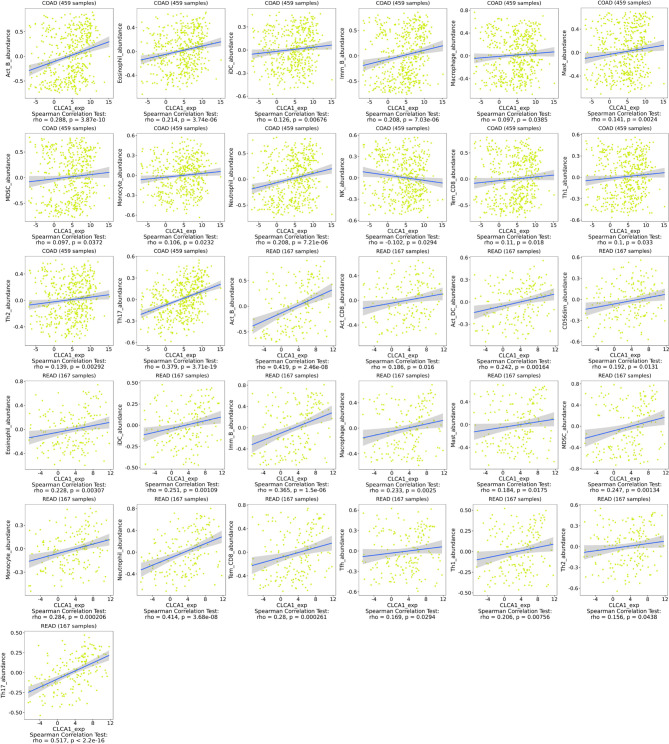
Relationship with immune infiltration. CLCA1 in colon cancer and rectal cancer.

We utilized TISIDB to explore the relationship between hub gene expression levels and lymphocyte levels in colon and rectal cancer. CLCA1 exhibited no relationship or only a weak relationship with immune infiltration (**Figure 9**). CLCA1 expression was closely correlated with Th17 (rho=0.379, P=3.71e-19) levels in colon cancer and with Act-B (rho=0.419, P=2.46e-08), ImmB (rho=0.365, P=1.5e-06), neutrophil (rho=0.414, P=3.68e-08), and Th17 (rho=0.517, P<2.2e-16) levels in rectal cancer.

### Validation of Protein Expression and Prognostic Value of CLCA1

**Figure 10:**
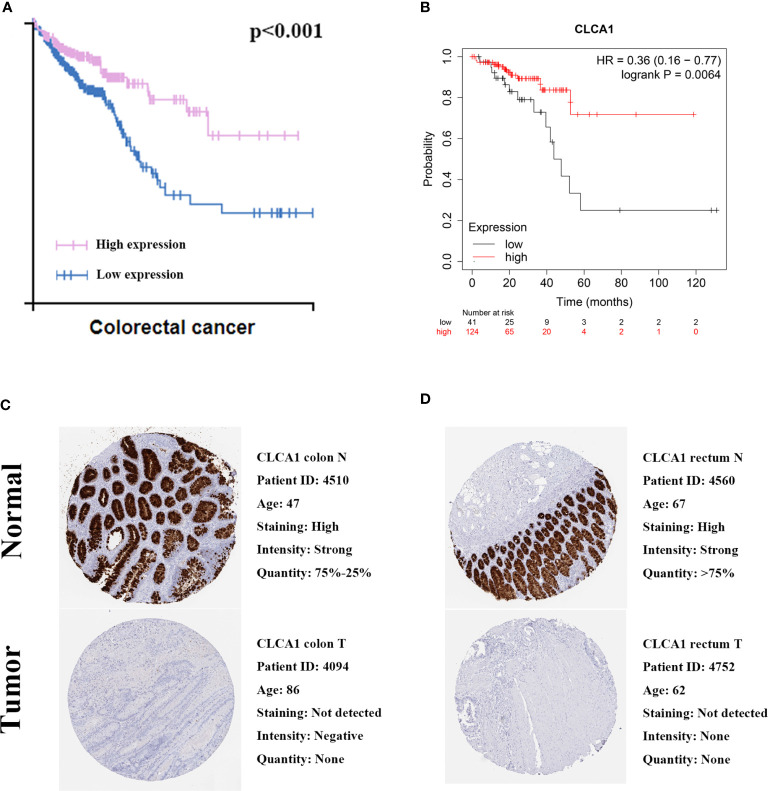
Validation prognostic value. **(A)** Prognostic value in The Human Protein Atlas. **(B)** Prognostic value in Kaplan-Meier Plotter. **(C)** Immunohistochemical in The Human Protein Atlas of colon. **(D)** Immunohistochemical in The Human Protein Atlas of rectum.

We utilized GEO datasets to valdiate the CLCA1,P value was >0.05 (**Supplementary Figure 2**). We also utilized The Human Protein Atlas and Kaplan-Meier Plotter database to validate the protein expression and prognostic value of CLCA1 (**Figure 10**). The protein expression in normal colon tissue was significantly higher than that in colon cancer tissue, and the same was true in the rectum. Both of the two databases showed the good prognostic value for CRC, the P value of The human protein atlas is <0.001 and KM plotter database is 0.0064.

## Discussion

CRC is the most frequently diagnosed gastrointestinal cancer (30), and the current colonoscopic diagnosis of CRC has limitations (31); therefore, identifying a significant biomarker for CRC is necessary. Gene microarrays were utilized to discover novel biomarkers or therapeutic targets for CRC (32). To our knowledge, our work is the first to combine RRA analysis of GEO datasets with WGCNA of TCGA datasets to explore the significant genes associated with CRC. Some diseases of the intestinal tract, such as intestinal polyps (33) and inflammatory bowel disease (34), can have symptoms similar to those of CRC and can also develop into cancer. To explore potential DEGs between tumor tissue and noncancerous tissue, we compared normal tissue, normal matched tissue and paratumor tissue with CRC tissue as control tissue. We integrated 10 datasets from GEO, TCGA-COAD and TCGA-READ and identified robust DEGs, such as SST (35), SLC26A3 (36), and SLC4A4 (37), which have been reported to be diagnostic biomarkers or therapeutic targets for CRC.

We used GO and KEGG enrichment analyses to explore the functions of the DEGs identified by overlapping the DEGs in the 3 datasets. GO analysis indicated that negative regulation of growth, bicarbonate transport, and transporter activity (38–40) were closely related to the development and growth of cancer; some KEGG pathways, such as nitrogen metabolism and retinol metabolism, were also linked to the pathogenesis of CRC. Nitrogen is an essential biomolecule in humans and regulates cellular metabolism, and retinol is a form of vitamin A closely related to immune functions (41, 42). Based on the results of the GO and KEGG enrichment analyses, the DEGs were closely associated with CRC occurrence and development.

Cytohubba can extract key sub-networks, and MCC is a newer algorithm of cytohubba. To identify the key genes among 129 DEGs, we utilized MCC to determine the top 20 hub genes (CLCA1, TMIGD1, SLC30A10, MT1F, MT1M, MT1G, MT1H, MT1E, GUCA2B, GUCA2A, SLC26A3, CLCA4, MS4A12, SI, SLC9A2, GCG, PYY, SST, SLC4A4, and SLC16A9). To explore the potential functions of the hub genes, we utilized R packages, ROC curves, K-M analysis, and GO and KEGG analyses. According to the results, all the hub genes were closely related to each other and had high diagnostic value, but only CLCA1 was associated with survival time. In addition, the hub genes were closely associated with the development of CRC.

To determine the hub genes significantly associated with overall survival, we utilized Cox proportional hazards regression analysis to develop a prognostic model. We explored each gene’s characteristics. Used ROC curve and random forest analysis to verify the model. The AUC values were high for 1-year, 3-year, and 5-year survival, all of which demonstrated the intermediate value of the prognostic model. Then, we calculated the risk score of each patient and divided the patients into a high-risk group and a low‐risk group. K-M risk survival analysis showed that the model can predict survival time. Then we utilized the random forest method to validate the prognostic model for CRC, which showed high prognostic value for CRC. The yielded the following values: accuracy = 79.3%, sensitivity = 85% which reflecting good prognositc value for CRC. Among the hub genes, only CLCA1 was associated with a good prognosis in CRC but their dignostic value is very high. In accordance with the expected results, the expression of CLCA1 protein was down-regulated in colorectal cancer tissues. To demonstrate the prognostic value of CLCA1, we conducted the external validation in three online databases and the results of The Human Protein Atlas and KMplotter showed that CLCA1 has a high prognostic value. The results were inconsistent with GEO database, and the unsatisfactory results of validation of GEO database may be related to the possible influencing factors such as sample size, experimental environment and methods.

Although CLCA1 has a high prognostic value for CRC, the mechanism of its influence is unclear. To further explore its characteristics, we analyzed differences in CLCA1 expression levels between tumor and normal tissues, across clinical stages, and across TNM stages. There was a large difference between tumor and normal tissues, but no significant differences were found across the different stages of CRC. This pattern indicates that CLCA1 levels decrease starting from the initial development of CRC and have diagnostic value at every stage of CRC. The characteristic expression of CLCA1 may provide a new perspective for exploring CRC at the gene level and serve as a useful diagnostic biomarker for CRC.

To explore the mechanisms of the hub genes in CRC, we utilized TISIDB and the R package “estimate” to assess immune infiltration and GSEA data of biological functions for CLCA1. TISIDB and the estimated score analysis indicated that CLCA1 had a weak relationship with lymphocyte expression and was expressed mainly in CRC cells. GSEA indicated that CLCA1 was enriched in “ascorbate and aldarate metabolism”, “butanoate metabolism”, “fatty acid metabolism”, “starch and sucrose metabolism”, and “valine, leucine, and isoleucine degradation”, suggesting that CLCA1 can influence CRC development and progression through different metabolic pathways. This result provides new insight into the mechanism and pathology of CRC.

In summary, we determined that CLCA1 could be used as a prognostic marker for CRC and correlated with immune infiltration. It may be a potential therapeutic target for CRC to improve the prognosis of patients. However, our work has some limitations. First, more work needs to be done on the pathogenic immune responses and gene expression in CRC cells to identify the mechanism linking the immune response with the development of CRC. Second, validation in GEO datasets is not ideal and pure bioinformatics analysis cannot well prove the prognostic significance of CLCA1 in colorectal cancer, in future research we will focus on large-scale population for further investigation. Furthermore, basic research needs to be done to verify our model and the regulatory mechanism *in vitro* and *in vivo*.

## Data Availability Statement

The datasets presented in this study can be found in online repositories. The names of the repository/repositories and accession number(s) can be found in the article/**Supplementary Material**.

## Author Contributions

F-ZW designed the research. Z-JW, S-WM, JL, and H-YS organized the data. J-NC, F-QZ, and ZL analyzed and visualized the data. F-ZW drafted the article. QL revised the paper. All authors contributed to the article and approved the submitted version.

## Funding

Key Project of National Key R & D Plan “Research on Prevention and Control of Major Chronic Non-Communicable Diseases” (No. 2019YFC1315705), China Cancer Foundation Beijing Hope Marathon Special Fund (No. LC2017L07), Medical and Health Science and Technology Innovation Project of the Chinese Academy of Medical Sciences (No. 2017-12M-1-006).

## Conflict of Interest

The authors declare that the research was conducted in the absence of any commercial or financial relationships that could be construed as a potential conflict of interest.

## Abbreviations

Act_B, Activated B cell; Act_CD4, Activated CD4 T cell; Act_CD8, Activated CD8 T cell; ACT_DC, Activated dendritic cell; CD56bright, CD56bright natural killer cell; CD56dim, CD56dim natural killer cell; DEG, differentially expressed gene; GEO, Gene Expression Omnibus; GO, Gene Ontology; GSEA, Gene Set Enrichment Analysis; iDC, Immature dendritic cell; Imm_B, Immature B cell; KEGG, Kyoto Encyclopedia of Genes and Genomes; Mast, Mast cell; MCC, Maximal Clique Centrality; MDSC, Myeloid-derived suppressor cell; Mem_B, Memory B cell; NK, Natural killer cell; NKT, Natural killer T cell; pDC, Plasmacytoid dendritic cell; ROC, Receiver Operating Characteristic; RRA, robust rank aggregation; Tcm_CD4, Central memory CD4 T cell; Tcm_CD8, Central memory CD8 T cell; TCGA, the Cancer Genome Atlas; Tem_CD4, Effector memory CD4 T cell; Tem_CD8, Effector memory CD8 T cell; Tfh, T follicular helper cell; Tgd, Gamma delta T cell; Th1, Type 1 T helper cell; Th17, Type 17 T helper cell; Th2, Type 2 T helper cell; TNM, Tumor Node Metastasis; Treg, Regulatory T cell; WGCNA, Weighted Gene Co-Expression Network Analysis.
