# Corrigendum: Differential Expression Analysis Revealing CLCA1 to Be a Prognostic and Diagnostic Biomarker for Colorectal Cancer

**DOI:** 10.3389/fonc.2020.634122

**Published:** 2020-12-18

**Authors:** Fang-Ze Wei, Shi-Wen Mei, Zhi-Jie Wang, Jia-Nan Chen, Hai-Yu Shen, Fu-Qiang Zhao, Juan Li, Zheng Liu, Qian Liu

**Affiliations:** Department of Colorectal Surgery, National Cancer Center/National Clinical Research Center for Cancer/Cancer Hospital, Chinese Academy of Medical Sciences and Peking Union College, Beijing, China

**Keywords:** CRC, diagnostic, biomarker, prognostic model, database

In the published article, there was an error in the author affiliation. Instead of “Department of Colorectal Surgery, National Cancer Center/Cancer Hospital, Chinese Academy of Medical Sciences and Peking Union Medical College, Beijing, China”, it should be “Department of Colorectal Surgery, National Cancer Center/National Clinical Research Center for Cancer/Cancer Hospital, Chinese Academy of Medical Sciences and Peking Union College, Beijing, China”.

The authors apologize for this error and state that this does not change the scientific conclusions of the article in any way. The original article has been updated.

