# Supplementary figures and images for: Differential Expression Analysis Revealing CLCA1 to Be a Prognostic and Diagnostic Biomarker for Colorectal Cancer
Source: Front Oncol. 2020 Oct 28;10:573295. doi: 10.3389/fonc.2020.573295 (PMC7673386; doi:10.3389/fonc.2020.573295)

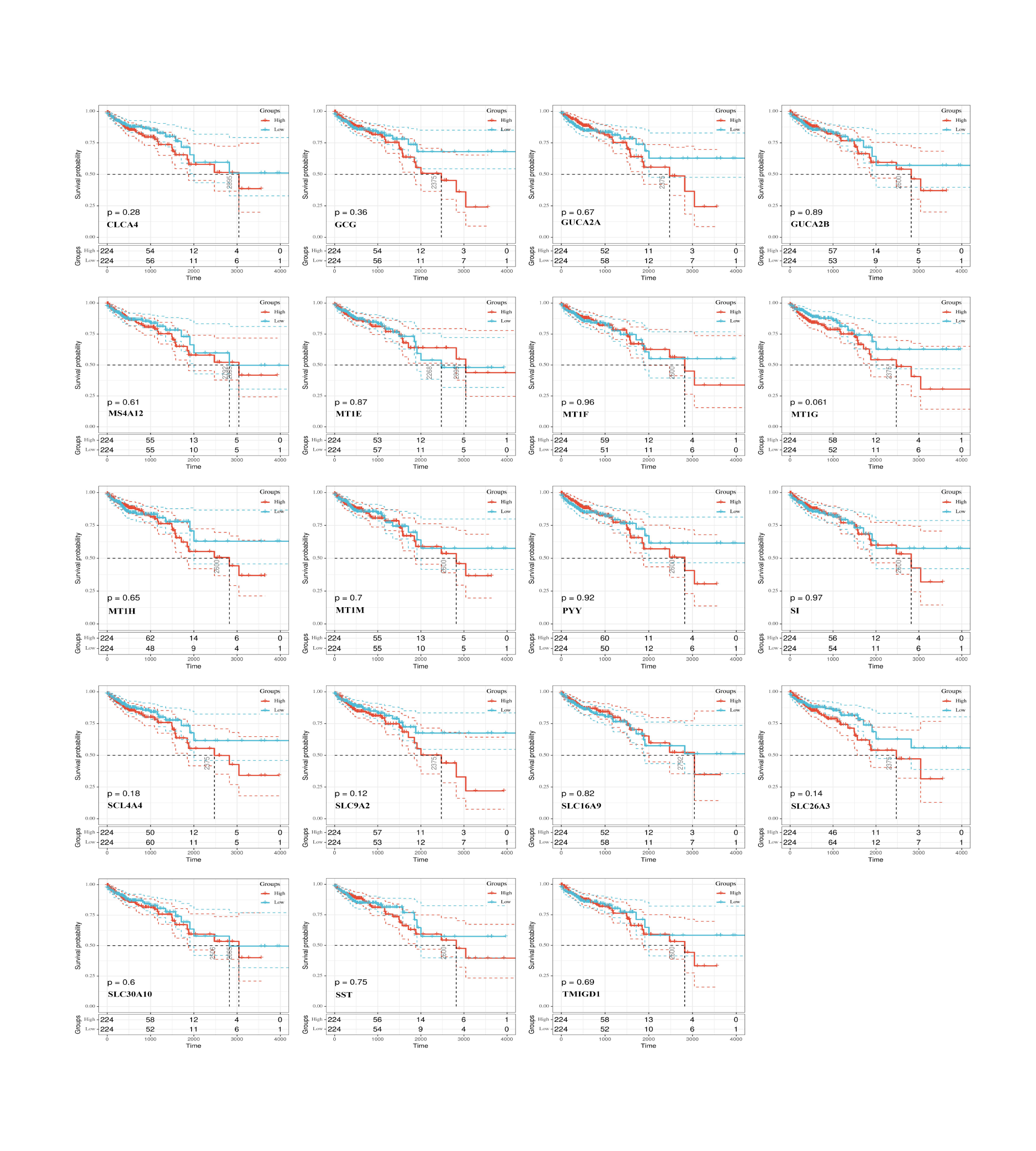

Supplement: Supplementary file 1 [file Image_1.tif]

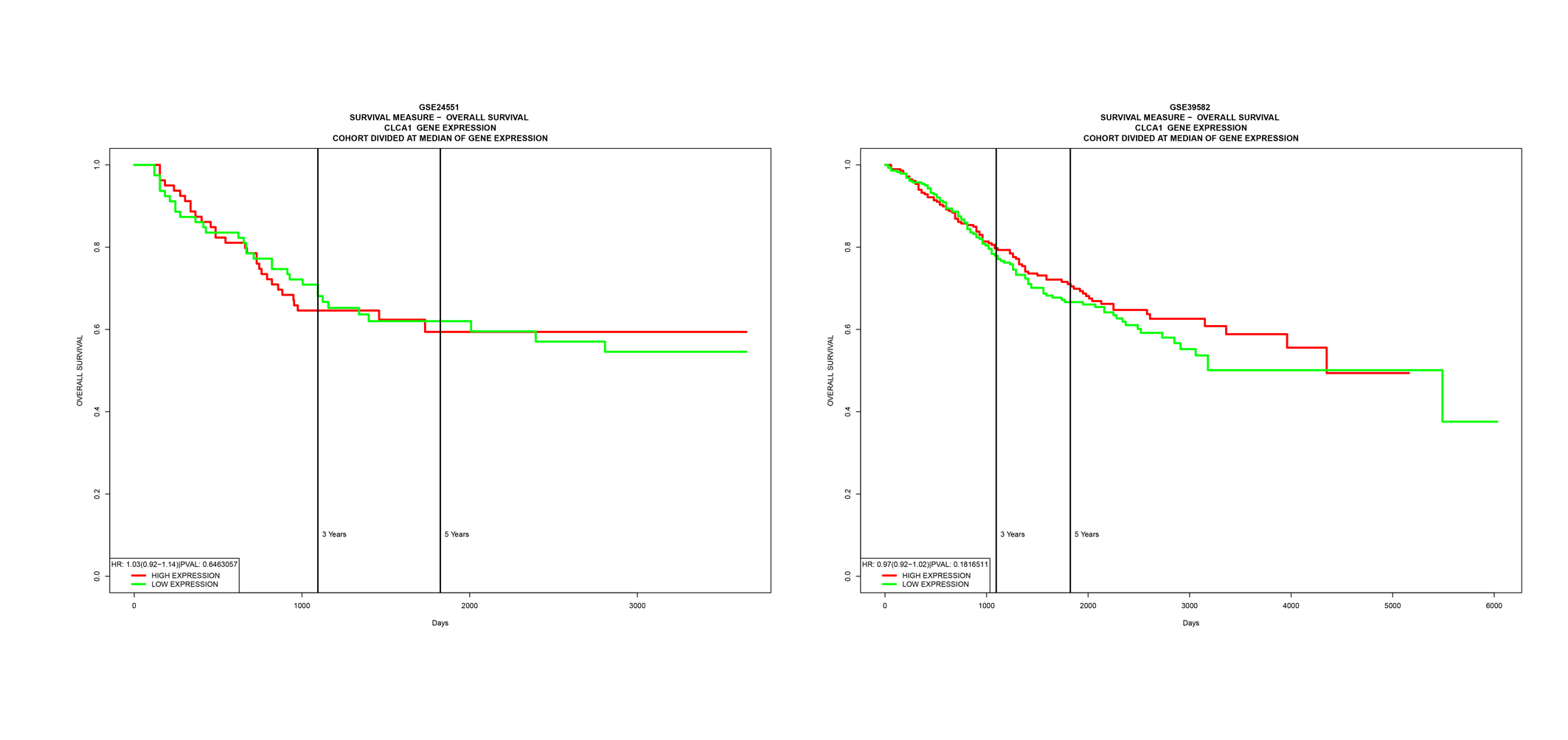

Supplement: Supplementary file 2 [file Image_2.tif]
